# Transcriptome analysis reveals new microRNAs-mediated pathway involved in anther development in male sterile wheat

**DOI:** 10.1186/s12864-018-4727-5

**Published:** 2018-05-08

**Authors:** Longqing Sun, Genlou Sun, Chenxia Shi, Dongfa Sun

**Affiliations:** 1 College of plant science & technology, Huazhong Agricultural University, Wuhan, 430070 Hubei China; 2 Biology Department, Saint Mary’s University, Halifax, Nova Scotia B3H 3C3 Canada; 3 Hubei Collaborative Innovation Center for Grain Industry, Jingzhou, 434025 Hubei China

**Keywords:** Wheat, Male sterility, Meiosis, miRNAs, Small RNA sequencing, Degradome, Tae-miR1127a, Tae-miR2275

## Abstract

**Background:**

337S is a novel bi-pole-photo-thermo-sensitive genic male sterile line in wheat, and sensitive to both long day length/high temperature and short day length/low temperature condition. Although the regulatory function of MicroRNAs (miRNAs) in reproductive development has been increasingly studied, their roles in pre-meiotic and meiotic cells formation of plants have not been clearly explored. Here, we explored the roles of miRNAs in regulating male sterility of 337S at short day length/low temperature condition.

**Results:**

Small RNA sequencing and degradome analyses were employed to identify miRNAs and their targets in the 337S whose meiotic cells collapsed rapidly during male meiotic prophase, resulting in failure of meiosis at SL condition. A total of 102 unique miRNAs were detected. Noticeably, the largest miRNA family was MiR1122. The target *CCR4-associated factor 1* (*CAF1*) of miR2275, a subunit of the Carbon Catabolite Repressed 4-Negative on TATA-less (CCR4-NOT) complex, contributes to the process of early meiosis, and was first identified here. Further studies showed that the expression of several pivotal anther-related miRNAs was altered in 337S at SL condition, especially tae-miR1127a, which may be related to male sterility of 337S. Here, we also identified a new member of SWI/SNF factors *SWI/SNF-*related matrix-associated actin-dependent regulator of chromatin subfamily A, member 3-like 3 (*SMARCA3L3*) targeted by tae-miR1127a, whose function might be involved in faithful progression of meiosis in male reproductive cells.

**Conclusion:**

The miRNA-target interactions of tae-miR2275-CAF1 and tae-miR1127a-SMARCA3L3 might be involved in regulating male fertility in 337S. Our results also implied that multiple roles for *SMARCA3L3* and *CAF1* in DNA repair and transcriptional regulation jointly orchestrated a tight and orderly system for maintaining chromatin and genome integrity during meiosis.

**Electronic supplementary material:**

The online version of this article (10.1186/s12864-018-4727-5) contains supplementary material, which is available to authorized users.

## Background

The improvement of wheat (*Triticum aestivum* L.) product is an important strategy to guarantee food security and solve the problem on feeding the population in China and many other countries with limited availability of cultivated land. Hybrid seed generated from heterosis utilization system has made a great contribution to food production. There are two well-known male sterility systems that have been developed for hybrid seed production: Cytoplasmic Male Sterile (CMS) and Photoperiod-Thermo-Sensitive Genic Male Sterile (PTGMS) [1]. The PTGMS system is considered to be more efficient than the CMS system for hybrid seed production because it can greatly simplify the procedure of hybrid [2].

The abnormality of the anther development is the main reason causing male sterility in plant. In flowering plants, anther development is an complex and precise biological process, including stamen meristem differentiation, generation of sporogenous cells and development of microspore mother cells, meiosis, microspore formation and maturation, and pollination [3], in which microsporocytes develop into mature pollen grains followed by twice mitotic divisions. Orderly, meiosis also involves in a series of complicated molecular events, including meiotic recombination, chromosome synapsis, cell cycle control, and chromosome distribution [4]. Meiotic recombination is one of the most important events during the early stage of meiosis, which is initiated by the generation of DNA double strand breaks (DSBs) [5]. To preserve genome integrity, cells have evolved several DSB repair mechanisms [6]. Basically, eukaryotic organisms employ two main mechanisms to repair DSBs: homologous recombination (HR) and non-homologous end-joining (NHEJ) pathways [7, 8]. The mammalian SWI/SNF chromatin-remodeling complex activated early during spermatogenesis is an essential meiotic factor for HR [9]. The *CAF1* encoding a conserved subunit of the CCR4-NOT complex is vital to meiotic progression, loss function of which resulted in sterility by blocking germ cell development at the pachytene stage of meiosis I in *C. elegans* [10].

Little is known about the roles of the SWI/SNF chromatin-remodeling complex and *CAF1* gene in plants. In *Arabidopsis*, a *SMARCA3*-Like chromatin remodeling factor regulates low-dosage UVB-dependent hypocotyl elongation [11]. Loss-function of both *AtCAF1a* and *AtCAF1b* reduced expression of pathogenesis-related genes and was more susceptible to pathogen infection [12]. However, the biochemical and physiological role of the *SMARCA* factors and *CAF1* were rarely reported in meiotic progression of plant.

Plant microRNAs are an abundance of non-coding 21–24 nt small RNAs. They regulate the expression of target genes by post-transcriptional degradation or translational repression [13]. Some conserved and classical miRNAs such as, miR156, miR159, miR160, miR164, miR167, miR396, miR5200, etc., were reported to be critical for reproductive development [14]. In maize, miR2118 and miR2275 were identified as the triggers for generating 21-nt and 24-nt reproductive phasiRNAs, respectively [15], which are primarily derived from *PHAS* loci of lncRNAs [16]. There are few reports about miRNA-mediated PTGMS in crops. In rice, phasiRNAs that were triggered by miR2118 and differentially accumulated in the panicles of Nongken 58S compared with its NIL line, especially under long-day conditions, caused male sterility of 58S [17]. miR167 and tasiRNA-ARF play roles in regulating the auxin-signaling pathway and possibly in response to cold stress, which linked with male sterility in the BS366, a thermo-sensitive genic male sterile (TGMS) lines of wheat, during early phase of anther development [18]. Further study found that miR964 and miR2186 regulatory pathway may participate in wheat male fertility transition [18]. Hence, it is of great application value and theoretical significance to explore the roles of miRNAs associated with the male sterility of PTGMS lines.

As an autumn-sown crop in the Hubei province of China, normal sowing time of wheat is from late October to early November. The wheat 337S line shows a high degree of male sterility if sown before September 30 or after November 30 every year, and it is fertile if sown in late October to early November at the same location. Further studies have proven that 337S is a male sterile line that is sensitive to both long day-length/high temperature and short day-length/low temperature condition, but it is fertile at normal planting time. These characteristics indicated that the fertility change of 337S line is due to the thermo-light condition of its cultivated environment, and 337S is a novel bi-pole-photo-thermo-sensitive genic male sterile (BP-PTGMS) line in wheat as described previously [19, 20]. In order to explore the mechanism of male sterility in 337S line at short day-length/low temperature condition, we characterized the roles of miRNAs and their targets in BP-PTGMS of wheat, using high-throughput small RNA and degradome sequencing analyses. The results revealed that genome-wide difference of genes expression and abnormity of the meiotic DSBs repair might be the main reasons leading to failure of meiosis and male sterility in 337S. Our results provided new knowledge on miRNAs regulating the fate of reproductive cell during early meiosis progression.

## Methods

### Plant materials and samples preparation

**Fig. 1:**
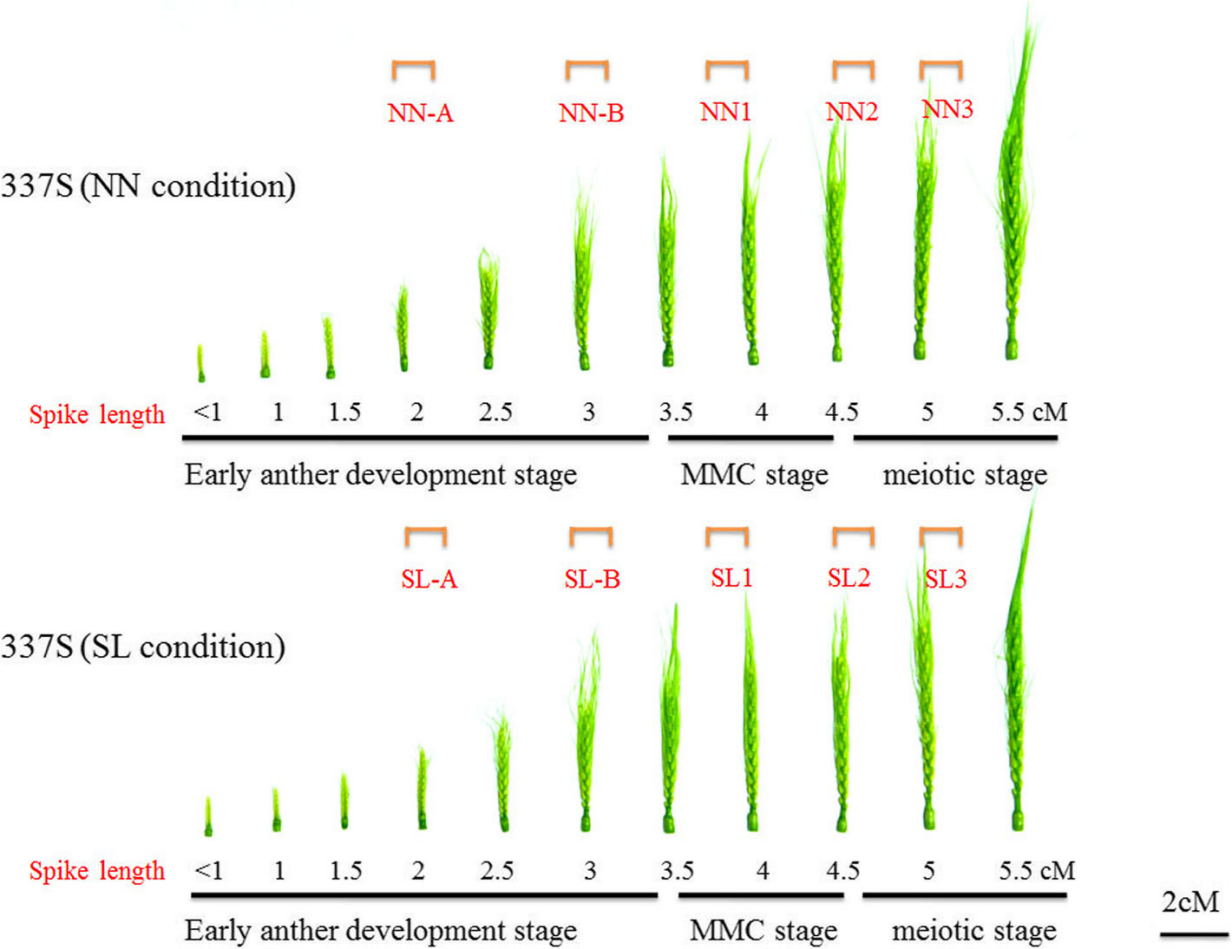
The spike phenotype from NN and SL plants of 337S at different developmental stages. The number under each spike was the length of spike without awn part. Bar = 2 cm. The image was obtained with a Nikon D90 camera

Wheat line 337S was cultivated in the experimental field of Huazhong Agricultural University, Wuhan, Hubei of China (N30°32′and E114°20′). The averaged temperature was 9.7 °C and day-length was 12.42 h during head development [21]. The planting time on Sept. 30 was considered as short day-length/low temperature (SL) environment, and Oct. 30 was considered to be normal day-length/normal temperature (NN) environment. The breeding material HZ09 and variety Huamai 2566 bred by our laboratory for cultivating in Hubei province were sown in the same plot as control. Each plot was 6 m long with three lines and inter-row spacing of 20 cm. During the anthesis developmental stages, young spikes of different lengths were recorded daily until flowering by measuring the space off lag leaf cushion and the length of the spike (without awn length) with scale ruler to confirm the pollen growth progress. The meiosis and microspore development process were observed using smear and squash techniques. The observation showed that the developmental progress of young spike in short day-length/low temperature was consistent with that of development in normal day-length/normal temperature condition (Fig. 1). Before sampling, NN and SL spikes of different lengths were marked by white tags, and then the samples of same developmental stage from the two environmental conditions were collected at the same time. Fifteen spikes were harvested for each sample, immediately frozen in liquid nitrogen and stored at − 80 °C for RNA extraction.

### Total RNA extraction

Total RNA was extracted from eight samples (NN1–1, NN1–2, NN2–1, NN2–2, SL1–1, SL1–2, SL2–1 and SL2–1) of 337S spikes, which included two developmental stage of anthers, each under two conditions (SL and NN) with two biological replicates, using TRIzol reagent (Invitrogen, Grand Island, NY, USA). RNA samples were sent to Novogene (Beijing, China) for library construction and sequencing.

### Library preparation and sequencing for small RNA

Four small RNA libraries consisted of two control (NN1 and NN2) and two treatments (SL1 and SL2), among which NN1 library was constructed using equal amount of RNAs from NN1–1 and NN1–2, NN2 from NN2–1 and NN2–2, SL1 from SL1–1 and SL1–2, SL2 from SL2–1 and SL2–2 A total amount of 3 μg total RNA per sample was used for the small RNA library. Libraries were generated using NEBNext Multiplex Small RNA Library Prep Set for Illumina (NEB, USA.), and sequenced on an Illumina Hiseq2500 platform.

### Bioinformatics analysis of sequencing data

To identify known and novel miRNAs, all raw reads were firstly subjected to the Illumina filter. After that, a certain range of length from clean reads of these small RNA tags were mapped to *Triticum aestivum* genome by Bowtie without mismatch to analyze their expression and distribution [22]. The tRNA, rRNA, small nuclear RNA (snRNA) and small nucleolar RNA (snoRNA) were BLASTN searched against the databases of Repeat Masker, Rfam (http://xfam.org/) and NCBI (http://www.ncbi.nlm.nih.gov/blast/Blast.cgi) and wheat genome. The filtered small RNA sequences were searched for conserved or known miRNA without mismatch. miRBase21.0 was used as reference, modified software mirdeep2 and srna-tools-cli were used to obtain the potential miRNA and draw the secondary structures. In addition, sRNA precursors containing classic hairpin structure of miRNA but not mapped to miRbase were used to predict novel miRNAs. The miREvo and mirdeep2 were integrated to predict novel miRNA through exploring the secondary structure, the Dicer cleavage site and the minimum free energy of the small RNA tags unannotated in the former steps. At the same time, custom scripts were used to obtain the identified known miRNA and novel miRNAs counts as well as base bias on the first position with certain length and on each position of all identified miRNA respectively. To map each unique small RNA to only one annotation, the following priority rule was used: known miRNA > rRNA > tRNA > snRNA > snoRNA > repeat > gene > NAT-siRNA > gene > novel miRNA > ta-siRNA. The miRNA expression levels were normalized by TPMs using the following formula: Normalized expression = mapped read count/Total reads*1000000 [23]. To identify miRNAs related to male abortion in 337S at the SL environmental condition, differential expression analysis of miRNA fold changes between two samples was performed using the DEGseq R package and adjusted using q value [24]. The threshold level of q-value (< 0.01) and absolute value of log_2_Ratio (≥1) were used to judge the significant difference of miRNAs expression levels. Known miRNAs were submitted to miFam (http://www.mirbase.org/ftp.shtml) database for family classification; novel miRNAs precursor was submitted to Rfam (http://xfam.org/) to search Rfam families. Target genes of known and novel miRNA were predicted using psRobot software.

### Degradome library construction and target identification

For the degradome sequencing, equal amounts of all NN (NN1–1, NN1–2, NN2–1 and NN2–2) or SL (SL1–1, SL1–2, SL2–1 and SL2–2) RNA samples used in small RNA sequencing were mixed together to generate two degradome libraries for searching the potential target of wheat miRNAs following the methods described previously [25]. The cDNA library was constructed and sequenced on Illumina Hiseq 2500 by Novogene (Beijing, China). Raw sequencing reads were obtained using Illumina’s Pipeline v1.5 software. After that, reads were mapped to the wheat genome to obtain cDNA sense and antisense tags. The tags mapped to cDNA or mRNA sequences were then used to predict cleavage sites. The pure reads with 20 and 21 nucleotides were used to identify potentially cleaved targets by PAREsnip (http://srna-workbench.cmp.uea.ac.uk/tools/paresnip/) and CleaveLand 3.0 (http://sites.psu.edu/axtell). The targets predicted were classified into five categories (0, 1, 2, 3 and 4) according to the previous study [26]. Based on the expression characteristics of the wheat transcriptome data, t-plots were built for the high-efficiency analysis of the potential miRNA targets. Finally, all candidate targets were used for function annotation and gene ontology (GO) analysis.

### Quantitative real-time reverse transcription-PCR

The total RNA was reverse-transcribed to miRNAs using the Mir-X™ miRNA First-Strand Synthesis and SYBR Kit (Clontech, CA, USA). The RNA was reverse transcribed to cDNA using the SuperScript III reverse transcriptase (Invitrogen). Quantitative real-time PCR was performed on the Bio-Rad CFX system (Bio-Rad Laboratories, California, USA) using *TaActin* as the internal control. The expression value of all genes was normalized by referring to sample NN1 or NN-A as ‘1’. The primers were listed in Additional file 1: Table S1.

### Histological analyses

Spikelets from the middle part of every spike were individually stripped from rachis with young glumes being removed from the spikelets. The anthers were then picked out using dissecting needle and fixed at room temperature for 24 h in FAA solution. After dehydration, the samples were infiltrated with Technovit 7100 resin and placed in an oven at a temperature series. Then blocks were trimmed and cut to 2 μm semi-thin sections using a fully motorized rotary microtome (Leica RM2265). The semi-thin sections of anther cross-sections were stained with 0.2% toluidine blue, and observed and photographed under the photomicroscope (DM2500, Leica, Wetzlar, Germany).

## Results

### 337S phenotype of different growth conditions from different sowing dates

The SL and NN plants of 337S were planting at different plots of the same experimental field (Additional file 2: Figure S1a and Additional file 3: Figure S2a). Significant difference among the 337S, HZ09 and Huamai 2566 plants at heading and flowering in SL early-sowing time was observed (Additional file 2: Figure S1b-d). 337S plants flowered about twenty days later than HZ09 and Huamai 2566 plants. When the 337S plants were at early booting stage, HZ09 and Huamai 2566 plants were at heading stage in SL condition (Additional file 2: Figure S1e). However, there was no significant difference on booting stage, heading date and flowering period among the 337S, HZ09 and Huamai 2566 plants in NN sowing-time (Additional file 3: Figure S2b-e).

Our previous study indicated that degeneration of microspore mother cells in meiosis I was the main reason resulting in 337S line sterile at SL condition [27]. The growth of anthers prior to the microspore stage was divided into 3 phases: early anther development stage (stage 1, from spikes differentiation to formation of sporogenous cell, spike length < 3.5 cm), microspore mother cell stage (stage 2, 3.5 cm ≤ spike length < 4.5 cm) and meiosis stage (stage 3, 4.5 cm ≤ spike length ≤ 5.5 cm) according to the length of the wheat spikes planted in the field. Morphologically, the vegetative development of spikes appears no obvious difference at different stages between SL and NN conditions (Fig. 1).

**Fig. 2:**
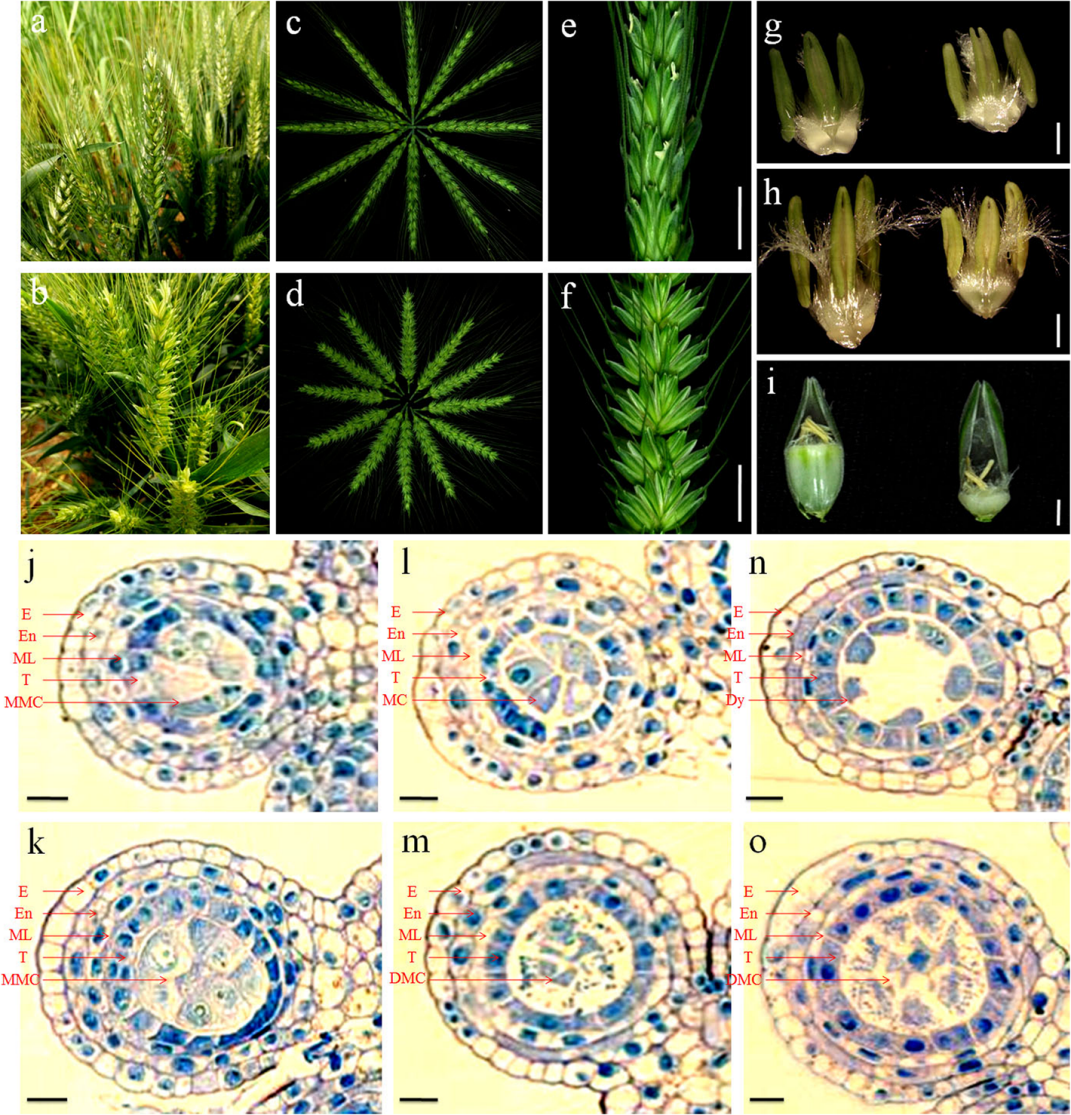
Phenotype comparison of 337S between NN and SL conditions. **a** The feature of 337S spikes planted at Oct. 30 that was defined as NN condition. **b** The feature of 337S spikes planted at Sept. 30 that was defined as SL condition. **c** The NN spikes of 337S at blooming stage. **d** The SL spikes of 337S at blooming stage. **e** A part of NN spike showed the elongated anthers at the pollination stage. Bar = 1 cm. **f** A part of SL spike showed glumes opening phenotype with a bigger angle at the pollination stage. Bar = 1 cm. **g** A NN anther (left) with slight yellow, smaller SL anther (right) at the mononuclear pollen stage. Bar = 1 mm. **h** Comparison between NN and SL anthers at late binuclear pollen stage. The SL anther was obvious shorter and yellow discolouration, while the NN anthers was still green. Bar = 1 mm. **i** Comparison between NN and SL spikelets after removing the lemma and palea, no visibly filled grains on SL plants at the filling stage. Bar = 2 mm. **j** and **k** Transverse section of single locule at microspore mother cell stage. Bars = 10 μM. **l** and **m** Transverse section of single locule at meiosis prophase stage, only cellular debris observed in the SL anther locule **m**. Bars = 10 μM. **n** and **o** Transverse section of single locule at dyad stage of meiosis, the meiotic cells further collapsed in the SL anther locule **o**. Bars = 10 μM. E, epidermis; En, endothecium; ML, middle layer; MC, meiocyte cell; MMC, microspore mother cell; DMC, degenerated meiocyte cell; Dy, dyad

Compared with NN plants of 337S, spikelets of SL plants were abnormal dehiscence (Fig. 2a-d). The glumes and lemmas of SL spikelets had a bigger angle than that of NN plants at full-bloom stage (Fig. 2e, f). The anthers of SL were shorter than those of NN plants, and SL anthers showed a slight yellow discolouration at mononuclear pollen stage and late binuclear pollen stage, while the NN anthers remained green (Fig. 2g, h). However, there was no significant difference of the pistil filaments between NN and SL plants (Fig. 2h). Unlike the normally grain filling on NN spikes, no visibly filled grain on SL plants at the filling stage was observed (Fig. 2i). To determine whether the SL plants could produce abnormal anthers leading to male sterility as reported previously [27], spike lengths about 3.9–4 cm, 4.5–4.6 cm and 5–5.1 cm corresponding to microspore mother cell stage (NN1/SL1) and meiosis stage (NN2/SL2, NN3/SL3) were collected from NN and SL plants for performing semi-thin sections, respectively (Fig. 1). Anthers transverse cross-section observation indicated that the anthers of SL1 plants developed normally until the microspore mother cell (MMC) stage (Fig. 2j, k). Histological results showed that the obvious aberration of SL2 anther occurred during meiosis, around the prophase stage, in which meiotic cells degenerated and collapsed rapidly, whereas the meiotic cells of NN2 anthers remained normally development (Fig. 2l, m). At the stage when the meiotic cells in NN3 anther produced dyads, the SL3 plant appeared similar phenotype to itself at prophase stage without any intact meiotic cells in anther chambers (Fig. 2n, o). These observations indicated that the first detectable sign of male sterility occurred at the meiotic prophase (MP) stage with degenerated meiocyte cell in SL2.

### Overview of the small RNA sequencing

**Fig. 3:**
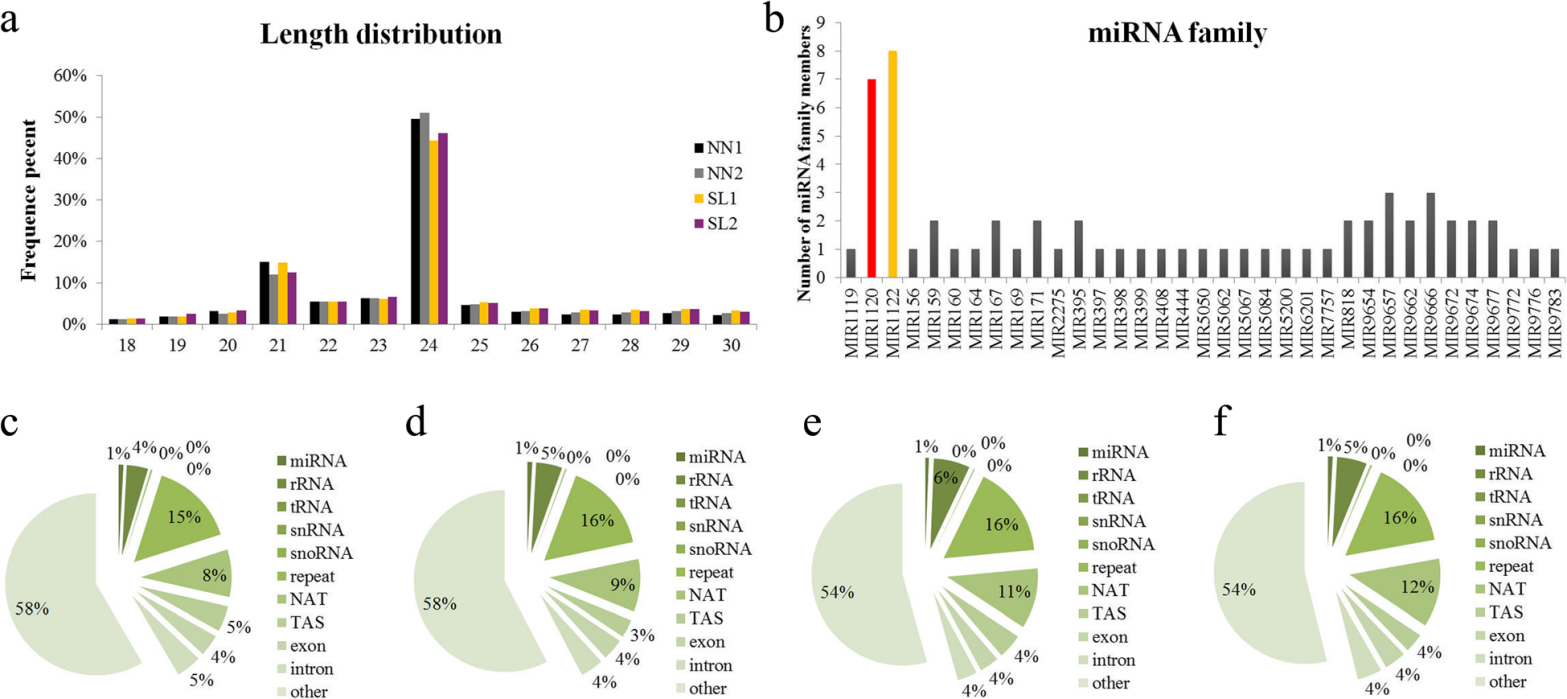
length distribution and family classification of small RNAs. **a** Sequence length distribution of total sRNA in the NN and SL samples. The 24-nt sRNA was the predominant one in each library. **b** Summary of conservative miRNAs belonged to the known miRNA families in wheat. The two largest numbers of these miRNA families were seven and eight, represented by the families of miR1120 and miR1122, respectively. **c** The classification of the total sRNAs annotation in NN1 library. **d** The classification of total sRNAs in NN2 library. **e** The classification of total sRNAs in SL1 library. **f** The classification of total sRNAs in SL2 library

In order to reveal the regulatory mechanism of SL abortion and explore new regulators for the anther development in wheat, small RNA and degradome sequencing was applied to identify critical genes leading to male sterility for 337S line. A total of 60,119,469 raw reads were obtained from four libraries; Of which 14,947,750 were generated from NN1 library, 14,290,542 from NN2, 14,998,252 from SL1, and 15,882,925 from SL2 library. After filter out reads of N% > 10%, reads with polyA/T/G/C, low-quality and adapter sequences, 14,597,462 (97.66%), 13,981,982 (97.84%), 14,635,455 (97.58%) and 15,584,410 (98.12%) clean reads were obtained from NN1, NN2, SL1 and SL2 libraries, respectively (Additional file 4: Table S2). The lengths of the majority of the sRNAs were 21~ 25 nt, in which the 24 nt sequences showed the highest enrichment among them, accounting for about half of the total reads, followed by 21 nt. A similar size distribution was observed for the length of 22 nt, 23 nt and 25 nt sRNAs in each library. Moreover, the frequency of 21 nt sRNAs in SL plants was consistent with the scale in NN plants at the same stage, such as 15.01 and 14.8% for NN1 and SL1, 12.04 and 12.55% for NN2 and SL2, respectively. However, the libraries of SL had higher abundance (49.6 and 51.02%) in 24 nt sequences than that of NN plants (44.38 and 46.06%) at both stages (Fig. 3a). sRNAs were annotated and grouped into several classes by aligning with known non-coding RNAs in the Rfam and NCBI database. Overall, the percentage of sRNA reads that matched for a specific group was similar among the four libraries. Nevertheless, interesting data was found from the unannotated reads between NN and SL plants. SL1 samples showed lower accumulation of unique unannotated reads than NN1 samples (54 and 58% of mapped sRNA for SL1 and NN1, respectively), likewise between SL2 and NN2 samples (Fig. 3c-f).

### Differentially expressed miRNAs between the SL and NN plants

The sRNAs with classic miRNA secondary structure for a length of at least 18 nt were aligned to all known plant miRNA sequences in miRBase21.0, 94 annotated known miRNAs were identified from the NN and SL libraries (Additional file 5: Table S3); Of which, 83 were from NN1 library, 84 from NN2, 89 from SL1, and 87 from SL2 library. Analysis of nucleotide bias at each position of miRNAs showed that the first nucleotide tended to be uracil (U) for each sample (Additional file 6: Figure S3). As shown in Fig. 3b, 62 known miRNAs were assigned to 35 known families, in which three fifths of the families contained a single member. Ten miRNA families were represented by only two members. Both MiR9657 and MiR9666 family contained 3 members. The two large numbers of these miRNA families were seven and eight, represented by MiR1120 and MiR1122 family, respectively (Fig. 3b). Prediction of novel miRNAs from the 4 sRNA libraries discovered the precursors for eight of the putative novel miRNAs, which were designated as Novel_01-Novel_08. All novel miRNAs were detected in the each of the four libraries. The precursor length of the novel miRNAs ranged from 57 to 292 nt, and were processed into 20 nt to 24 nt miRNAs (Additional file 7: Table S4). The expression level of each miRNA was normalized by TPM. More than 76% of TPMs for each of the four libraries were more than 60 (Additional file 8: Table S5).

**Fig. 4:**
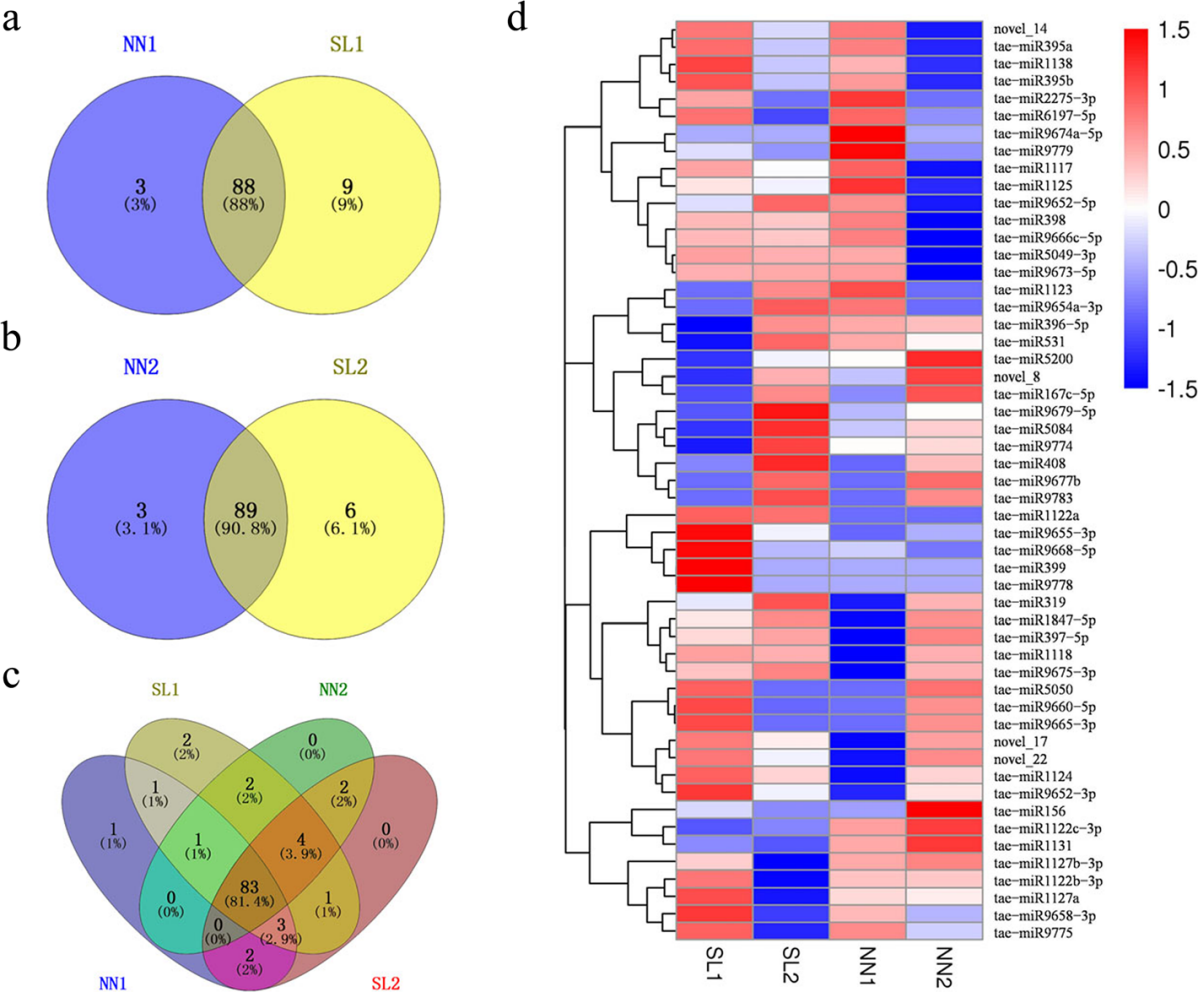
Venn charts of miRNA expression in four libraries and cluster analysis of differentially expressed miRNA. **a** Specific and commonly expressed miRNAs between NN1 and SL1 libraries. **b** The specific and common miRNAs identified from the two libraries (NN2 and SL2). **c** Venn distribution of differentially expressed miRNAs among the four libraries. **d** Hierarchical cluster analysis of differently expressed miRNAs in the four libraries. Red indicated up-regulation; blue indicated down-regulation

Comparative analysis of 102 unique miRNAs (94 known and 8 novel miRNAs) showed that 88 and 89 miRNAs overlapped between MMC stage and MP stage, respectively (Fig. 4a, b). Compared to NN samples, 9 and 6 specific miRNAs were detected in the SL samples at stages of MMC and MP, respectively (Fig. 4a, b). Two microRNAs, tae-miR399 and tae-miR9778, were specifically expressed in SL1 (Fig. 4c). One microRNA, tae-miR9674a-5p was only identified in NN1. However, these three sample-specific miRNAs were expressed at low levels (Additional file 9: Table S6). In addition, 83 miRNAs were common in all NN and SL samples (Fig. 4c). The heatmap showed 53 miRNAs differentially expressed among the four samples, including 52.1% (49 of 94) conserved miRNAs and 50% (4 of 8) novel miRNAs (Fig. 4d). 27 miRNAs were significantly differentially expressed in the sample of SL1 in comparison with the NN1 (control) at MMC stage, and 25 miRNAs were significantly differentially expressed between the SL2 and the NN2 (control) at MP stage (Additional file 10: Figure S4, Additional file 11: Table S7 and Additional file 12: Table S8). There were 10 common miRNAs across these two comparisons. Thus, total 42 significantly differentially expressed miRNAs were found in both MMC and MP stages together (SL1 vs NN1 and SL2 vs NN2) (Additional file 10: Figure S4), 60 miRNAs showed no differential expression at both MMC and MP stages (Additional file 13: Table S9–1). The 42 differentially expressed miRNAs were divided into 5 categories based on expression pattern. Only tae-miR1122a was upregulated in the both two comparisons (Additional file 13: Table S9–2), while tae-miR1122c-3p and tae-miR5200 were downregulated in these two comparisons (Additional file 13: Table S9–3). Seven miRNAs showed opposite expression patterns between the two comparisons (Additional file 13: Table S9–4). 17 miRNAs exhibited significantly differential expression at MMC stage (Additional file 13: Table S9–5), 15 miRNAs showed significantly differentially at MP stage (Additional file 13: Table S9–6).

**Fig. 5:**
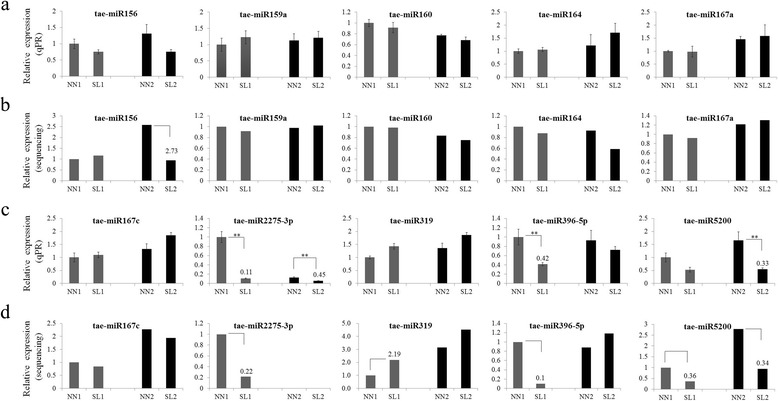
Fold-change of conserved and classical miRNA proved to be essential for reproductive development in 337S. **a** and **c** qRT-PCR data. The transcript level of each gene was normalized to NN1. The fold change normalized ≥ 2 or ≤ 0.5 were represented by numerical value. Error bars indicated s.d. based on three biological replicates (**P < 0.01, Student’s *t*-test). **b** and **d** Transcriptome sequencing data. The expression value of all genes was normalized to NN1. The fold change normalized ≥ 2 or ≤ 0.5 were used for judging significant differentially expressed genes in sequencing data and represented by numerical value

qRT-PCR was performed to validate the sequencing data. Based on the data analysis for each category above, the miRNAs which had been confirmed to be conservative in reproductive development of plant and two novel miRNAs were selected for qRT-PCR analysis. The results showed that the expression patterns of qRT-PCR for these differentially expressed miRNAs were consistent with the expression trend of RNA-Seq data, indicating high reliability of the sequencing (Fig. 5 and Additional file 14: Figure S5). According to previous studies, miR156, miR159, miR160, miR164, miR167, miR319, miR396 and miR5200 were mainly involved in floral development [14, 28]. No obvious expression changes of tae-miR159a, tae-miR160, tae-miR164, tae-miR167a and tae-miR167c were found between SL1 and NN1, and between SL2 and NN2 samples. tae-miR156 and tae-miR319 did not show two-fold change as RNA-seq data did. Sequencing data indicated the expression levels of tae-miR2275-3p, miR396-5p and tae-miR5200 were significant difference between NN1 and SL1 plants, and downregulated in SL1 plants. tae-miR2275-3p and tae-miR5200 were also extremely suppressed in SL2 compared to NN2 plants (Fig. 5).

### Identification of miRNA targets through degradome sequencing

From degradome sequencing, more than 1*10 million raw reads from each library were obtained. After removing the reads < 15 nt and 3′ adaptor, 9,829,031 and 9,421,684 transcript reads from SL library and NN library were mapped to wheat genome, respectively (Additional file 15: Table S10). The predicted targets were classified into five categories (0, 1, 2, 3 and 4). The targets with category 0 were evaluated as the most significant.

**Fig. 6:**
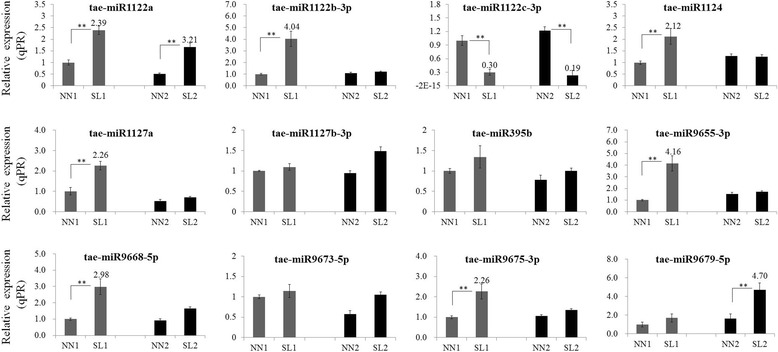
Fold-change of the special miRNA in each library based on the qRT-PCR and sRNA sequencing. The transcript level of each gene was normalized to NN1. The fold change normalized ≥ 2 or ≤ 0.5 were represented by numerical value. Error bars indicated s.d. based on three biological replicates (**P < 0.01, Student’s *t*-test)

According to the expression characteristics of the wheat transcriptome data, t-plots were built for the high-efficiency analysis of the identified miRNA targets. As a result, a total of 643 transcripts targeted by 53 known miRNAs were obtained from transcriptome analysis. The tae-miR1127b-3p targets the highest number of 124 annotated and unknown transcripts. Nevertheless, no candidate target was validated for the novel miRNAs identified here. Further, total of 257 transcripts targeted by 20 miRNAs that were significant differentially expressed at MMC or MP stages were screened out from degradome data (Additional file 16: Table S11). Then, 12 miRNAs with TPMs > 25 in at least one sample in Table S9–5 and Table S9–6 together with tae-miR1122a in Table S9–2, tae-miR1122c-3p in Table S9–3 and tae-miR1122b-3p in Table S9–4 (marked by *) were validated by qRT-PCR. qRT-PCR showed that tae-miR1122a, tae-miR1122b-3p, tae-miR1122c-3p, tae-miR1124, tae-miR1127a, tae-miR9655-3p, tae-miR9668-5p, tae-miR9675-3p and tae-miR9679–5p were two-fold differentially expressed between SL1 and NN1, or between SL2 and NN2 (Fig. 6). However, the targets of tae-miR1124, tae-miR9655-3p and tae-miR9668-5p were unknown (Additional file 17: Table S12). Therefore, the 6 candidate miRNAs tae-miR1122a, tae-miR1122b-3p, tae-miR1122c-3p, tae-miR1127a, tae-miR9675-3p, tae-miR9679–5p together with tae-miR2275-3p were selected to further dissect their functions in wheat reproductive development. In addition, no target was identified for both tae-miR396-5p and tae-miR5200 from degradome data.

**Fig. 7:**
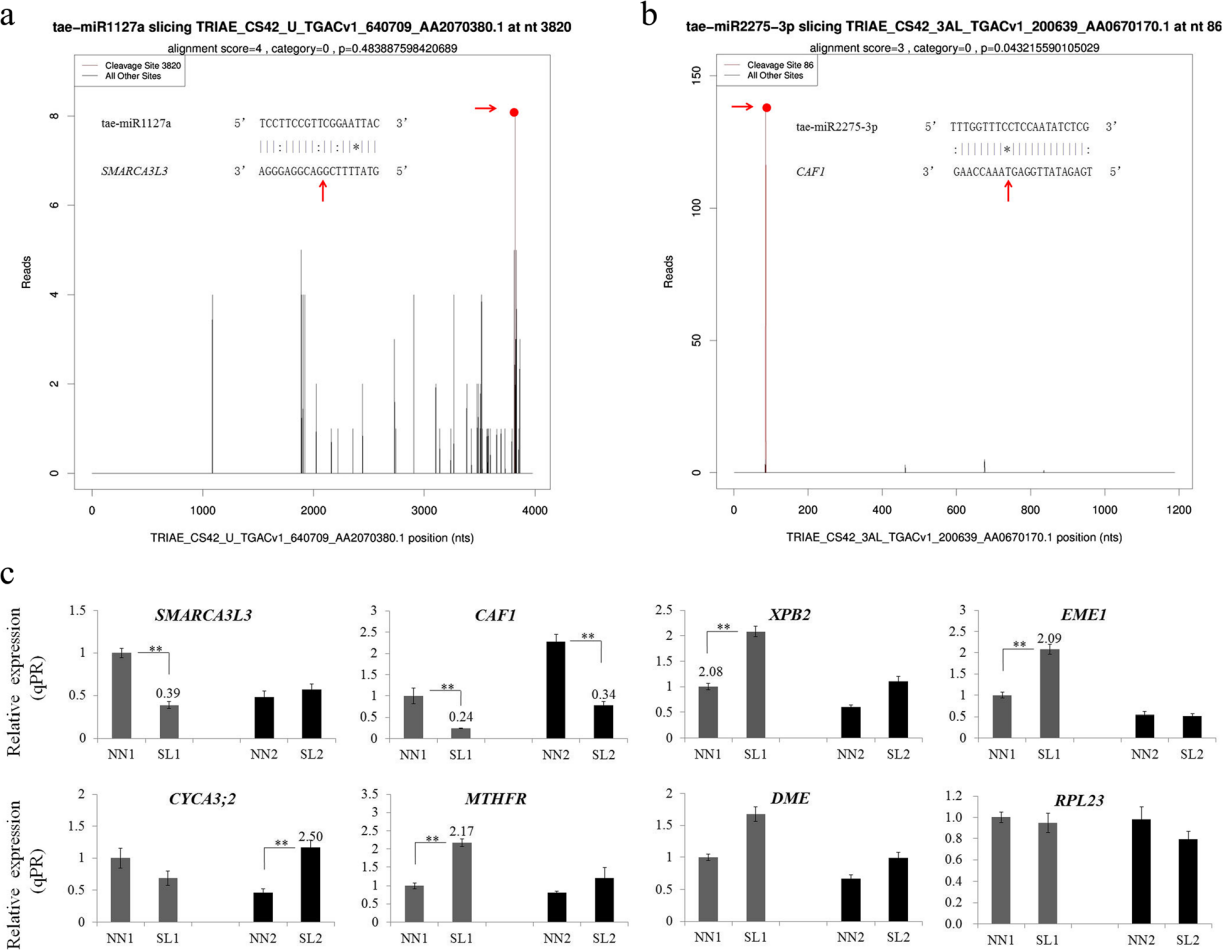
T-plots of representative miRNA targets and expression analysis of target genes identified from degradome sequencing. **a** and **b** The T-plots of *SMARCA3L3* and *CAF1* targeted by tae-miR2275-3p and tae-miR1127a. The T-plots showed the distribution of the degradome tags from the target gene sequence. miRNA cleavage was represented by red lines with spots. Cleavage sites for SMARCA3L3 and CAF1 were at 86 nt and 3820 nt, respectively. **c** The relative expression of selected targets from degradome data for several key miRNAs, *CAF1* of tae-miR2275-3p, *SMARCA3L3* of tae-miR1127a, *XBP2* and *EME1* of tae-miR1122c-3p, *CYCA3;2* of tae-miR1122b-3p, *MTHFR* of tae-miR1122a, *RPL23* of tae-miR9675-3p and *DME* of tae-miR9679–5p. The transcript level of each gene was normalized to NN1. The fold change normalized ≥ 2 or ≤ 0.5 were represented by numerical value. Error bars indicated s.d. based on three biological replicates (**P < 0.01, Student’s *t*-test)

Our degradome data showed a new and remarkable counterpart gene of mammalian SWI/SNF chromatin-remodeling complex, which may be associated with meiotic DSB in 337S. *SMARCA3L3* was the only target belonging to the cleavage sites classified for category = 0 (Fig. 7a) from all of the 14 genes targeted by tae-miR1127a (Additional file 16: Table S11). Noteworthy, the expression levels of *SMARCA3L3* was negatively correlated with that of tae-miR1127a, which was significantly downregulated in SL1 compared with NN1 at MMC stage (Fig. 7c). The expression of *XPB2* [29], a DNA repair helicase targeted by tae-miR1122c-3p, was highly upregulated in SL samples at both MMC and MP stages (Fig. 7c), implying that *XPB2* as a DNA damage detection recognizer may be induced to repair DNA in sterile plants of 337S. *EME1,* another target gene of tae-miR1122c-3p, is required for generating meiotic crossovers by resolving the double Holliday junction in fission yeast [30], its expression pattern was also changed in SL1 (Additional file 18: Figure S6). Moreover, previous studies showed that the *CAF1* encoding a conserved subunit of the CCR4-NOT complex was vital to meiotic progression [10]. Our degradome data revealed eight genes targeted by tae-miR2275-3p, three are *CAF1* homologs in the category 0 (Fig. 7b). Interestingly, the *CAF1* was dramatically suppressed in SL compared with NN at both MMC and MP stages (Fig. 7c). In addition, *cyclin A3;2* (*CYCA3;2*) [31], the cell cycle regulator, targeted by tae-miR1122b-3p, showed significantly differential expression at MP stage (Fig. 7c). The expression levels of *DEMETER* (*DME*) with DNA glycosylase activity for removing 5mC [32] targeted by tae-miR9679–5p were increased in SL plants (Fig. 7c). Methylenetetrahydrofolate reductase (MTHFR) that is the methyl donor for numerous cellular reactions [33] targeted by tae-miR1122a, was also upregulated in SL compared to NN plants. Furthermore, *60S ribosomal protein L23* (*RPL23*) as the unique target gene of tae-miR9675-3p [34] and *Defective in Anther Dehiscence1* (*DAD1*) [35], one of the targets of tae-miR1127b-3p, showed no obvious differential expression changes in SL plants (Additional file 19: Figure S7). The conserved miRNA-target interaction previously proved to be essential for floral development in plant, like tae-miR156-SPL17, tae-miR159a-GAMYB, tae-miR160-ARF18, tae-miR164-CUC2 and tae-miR167a-ARF12, showed very little changes in expression between SL and NN plants at MMC and MP stages (Additional file 19: Figure S7).

**Fig. 8:**
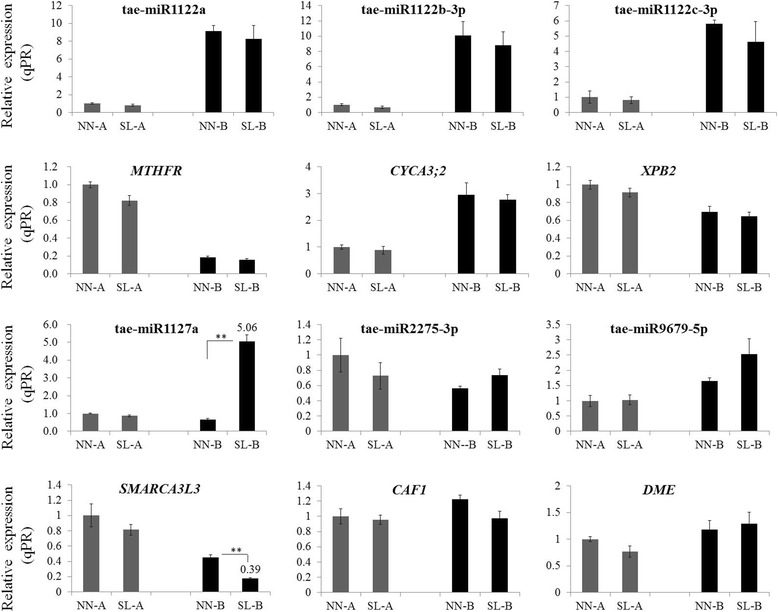
The expression profiles of miRNAs and their targets. Expression changes of miRNA and their target genes at early anther development stages. Samples were collected from normal day-length/normal temperature (NN-A/NN-B) and short day-length/low temperature (SL-A/SL-B) condition. The transcript level of each gene was normalized to NN-A. The fold change normalized ≥ 2 or ≤ 0.5 were represented by numerical value. Error bars indicated s.d. based on three biological replicates (**P < 0.01, Student’s *t*-test)

To further determine which miRNA-target interaction of tae-miR2275-CAF1, tae-miR1127a-SMARCA3L3, tae-miR1122c-XPB2, tae-miR1122c-EME1, tae-miR1122b-CYCA3;2, tae-miR9679-DME or tae-miR1122a-MTHFR is the vital and dominant determiner for regulating male sterility in 337S. Their expression patterns were analyzed in the stages prior to MMC. Spike lengths ranging from 2 to 2.1 cm and 3–3.1 cm corresponding to the early anther developmental stages were collected from normal day-length/normal temperature (NN-A/NN-B) and short day-length/low temperature (SL-A/SL-B) environment conditions (Fig. 1). Only one pair tae-miR1127a-SMARCA3L3 showed significant difference in both microRNAs and targets between SL-B and NN-B at early anther developmental stage. In addition, only the expression levels of *SMARCA3L3* and *CAF1* were negatively correlated with expression levels of tae-miR1127a and tae-miR2275-3p between SL-B and NN-B plants, respectively (Fig. 8). Therefore, the interactions of tae-miR1127a-SMARCA3L3 and tae-miR2275-CAF1 might be involved in regulating the male reproductive development in the 337S.

## Discussion

### Classical and conserved miRNAs participate in reproductive development of wheat

There are several conserved miRNAs that have been reported to be essential for reproductive development in plants, including miR156/7, miR159, miR160, miR164, miR165/166, miR167, miR169, miR172, miR319 and miR396 [14]. In this study, miR156, miR159, miR160, miR164, miR167, miR319 and miR396 were identified from our data. Moreover, miR2275 cleaving the precursor RNA to trigger the biogenesis of phasiRNAs in maize anther development at meiosis stage [15], were also found. However, only tae-miR2275-3p expressed significant difference between SL and NN plants as both miRNA-seq and qRT-PCR data indicated (Fig. 5). No target of tae-miR396-5p was detected from the degradome library. tae-miR5200 was previously identified as a Pooideae-specific microRNA that is expressed in leaves, and its overexpression severely delayed flowering time in *B. distachyon* transgenic plants [36]. However, in our study the phenotype of late-flowering was observed for SL plants, in which the tae-miR5200 expression was suppressed in both two anther development stages (Fig. 5). In addition, we found that tae-miR5200 expressed in reproductive tissues. How tae-miR5200 mediates late-flowering in 337S at SL condition still needs more evidence.

### The families of MiR1120 and MiR1122 are required for early anther development

In this study, a total of 94 known miRNAs were identified, two largest families were MiR1120 and MiR1122. MiR1120 consisted of 7 members (tae-miR1120a, tae-miR1120b-3p, tae-miR1120c-5p, tae-miR1121, tae-miR1122c-3p, tae-miR1137a and tae-miR1137b-5p). MiR1122 consisted of 8 members (tae-miR1122a, tae-miR1122b-3p, tae-miR1127a, tae-miR1127b-3p, tae-miR1128, tae-miR1133, tae-miR1135 and tae-miR1136). Only tae-miR1122c-3p of MiR1120 family, tae-miR1122a, tae-miR1122b-3p and tae-miR1127a of MiR1122 family showed significant differentially expression between SL and NN plants. Surprisingly, a microRNA tae-miR1127b-3p that targets 124 annotated and unknown transcripts did not show significant differentially expression between SL and NN plants (Fig. 6), especially during critical period of MMC stage. It might be considerable importance for reproductive development, but have little responsibility for early anther abortion in 337S. None targets for the 8 novel miRNAs were detected to have the cleavage signature from degradome data. tae-miR1122a, tae-miR1122b-3p, tae-miR1122c-3p and tae-miR1127a showed significantly differentially expression at MMC stage in SL1 plants (Fig. 6). However, the cell death appeared at MP stage in SL2 anthers (Fig. 2m), suggesting that the differentially expression might occur prior to phenotype differentiation. More potentially, the expression changes in these miRNAs might cause meiotic cells collapsing in SL anthers that directly lead to failure of meiosis and male sterility. Whereas, miR1122 and miR1127 were mainly involved in response to drought stress and dehydration stress [37–39]. Here, we found that tae-miR1122a, tae-miR1122b-3p, and tae-miR1127a of MiR1122 family might be effectively involved in modulating the process of meiosis.

### tae-miR2275 might be involved in generating 24-phasiRNAs in wheat

miR2275 and miR2118 were identified as the triggers for generating 21-nt and 24-nt phasiRNAs at meiosis and premeiotic stage, respectively [15]. However, tae-miR2118 was expressed with low abundance in wheat anthers development from the MMC stage to meiosis stage in previous report [18], here it was also undetectable at MMC and MP stage. Therefore, as a conservative microRNA in monocotyledon plants (Additional file 20: Table S13), tae-miR2275-3p may play a dominative role for meiosis in wheat. As shown in Fig. 5, the expression level of tae-miR2275-3p in SL plants was much lower than that in NN plants at MMC stage and MP stage. In the meantime, as mentioned in Fig. 3a, SL plants had lower abundance of 24 nt small RNAs than NN plants, but here we lacked the measurement data for 24-phasiRNAs identification. Thus, more work is needed to test if tae-miR2275-3p may be also involved in generating 24-phasiRNAs in wheat in future. Previous results indicated that the germline-specific *Argonaute* MEL1 (MEIOSIS ARRESTED AT LEPTOTENE1) protein, a rice AGO, regulated the cell division of premeiotic germ cells. Chromatin modification is altered and meiosis is arrested at early prophase I in *mel1* mutant meiocytes [40]. *MEL1* AGO participated in the miR2118-dependent, and the DCL4-dependent pathways is required for biogenesis 21-nt small RNAs [41]. But AGOs associated with biogenesis of 24-nt phasiRNAs remain unknown [42]. The microRNA tae-miR9652-5p showed differentially expression in our miRNA-seq data, its target gene was not identified from degradome sequencing. However, total 14 genes were predicted to be targets of tae-miR9652-5p using PsRobot software, 8 of them were annotated as the homologues of *OsMEL1* in rice (Additional file 21: Table S14). In this study, the expression of *MEL1* was significantly suppressed in SL1 and SL2 compared to NN1 and NN2, which was negatively related with expression levels of tae-miR9652-5p. Moreover, it was initially slightly down-regulated in SL-B (Additional file 22: Figure S8). Thus, the downregulation of *MEL1* might be related to the failure of meiosis and male sterility of 337S at SL condition. Overall, tae-miR2275-3p might be also involved in biogenesis of 24-nt phasiRNAs that might be associated with the *MEL1* gene targeted by tae-miR9652-5p at premeiotic or meiotic stages in wheat.

### The failure of meiotic DSB repair might arise and lead to meiotic cell death in 337S

In male sterile line of 337S, abortion occurred at meiotic prophase I. During meiosis, recombination between homologous chromosomes is initiated by the formation of programmed DSBs [43] that are introduced along chromosomes to promote homolog recombination and pairing at the first meiotic division [44]. DSBs are highly toxic lesions that can drive genetic instability [6]. Failure to repair a DSB has deleterious consequences, including genomic instability and cell death [7]. The mammalian SWI/SNF chromatin-remodeling complex which was activated early during spermatogenesis is an essential meiotic factor for HR [9]. The loss function of chromatin-remodeling factor INO80 led to abnormal behavior of meiosis which was represented by a failure of repairing DSBs during HR at the early stages of meiotic prophase I [45]. In addition, the catalytic subunit of SWI/SNF complex, BRG1, is required for HR, and the loss-function of this subunit results in arrest at the pachytene stage of meiosis I [46].

Loss function of *CAF1* resulted in sterility of *C. elegans* by blocking germ cell development at the pachytene stage of meiosis I [10]. *mCAF1*^−/−^male mice are sterile with a phenotype of complete disappearance of germ cells, indicating that *CAF1* is an essential factor for spermatogenesis [47]. In addition, depletion of *CNOT1,* that is one of the non-catalytic subunits for *CCR4–NOT* complex in mammals induces apoptotic cell death [48]. Further, CCR4–NOT complex is required for replication stress or DNA damage in *Saccharomyces cerevisiae* [49]*.* A novel role of this multiprotein complex has been identified in maintaining heterochromatin integrity at subtelomeres and heterochromatin islands in fission yeast [50]. Interestingly, the *SWI/SNF* chromatin-remodeling complex is essential to remodeling and efficient inducing RNR genes after DNA damage that is dependent on the *CCR*4–*Not* complex [49]. Overall, both SWI/SNF complex and CCR4–NOT complex may be required for DNA damage response in eukaryotic cells, loss-function of which can lead to genomic instability.

Thus, SWI/SNF complex and CCR4–NOT complex play vital roles in regulating the cell fate and DNA repair. However, the relationship between SWI/SNF complex and CCR4–NOT is not clear. In this study, histological results showed that the meiotic cells in SL anthers died and degenerated rapidly at the stage of meiotic prophase I (Fig. 2m). Moreover, the expression of SWI/SNF chromatin-remodeling factor *SMARCA3L3* and CCR4–NOT subunit *CAF1* were both suppressed in SL1 plants before meiosis (Fig. 7c). Further, the *XPB2*, a target of tae-miR1122c-3p, was responsible for DNA repair pathway as a key member of the human *TFIIH* complex [51], whose homologue gene *AtXPB* from *Arabidopsis thaliana* was also revealed to be involved in DNA repair [29]. *EME1* as another target gene of tae-miR1122c-3p is required for generating meiotic crossovers by resolving the dHJ in yeast [30]. In our study, both expression patterns of *XPB2* and *EME1* were also altered in SL1 plants (Fig. 7c and Additional file 18: Figure S6). However, the expression of *SMARCA3L3* showed significant repression and *CAF1* showed slight reduction in SL-B plants, which was prior to *XPB2* and *EME1* (Fig. 8). *SMARCA3L3* and *CAF1* might take a dominating role for meiotic DNA repair in 337S. Therefore, tae-miR2275-3p that targets *CAF1* and tae-miR1127a that targets *SMARCA3L3*, might be relative to the regulation of chromatin integrity in wheat. It also implied that the process of meiotic DSBs repair might be failed during meiosis I in SL plants due to expression changes of these genes, and then leading to abortion in 337S at SL condition.

### The homeostasis of genome-wide genes expression is quite important at premeiotic and meiotic stages

As mentioned above, *SMARCA3L3* and *CAF1* might be essential for meiosis DSBs process in 337S of wheat. Moreover, the CCR4–NOT complex is a global regulator that conserved from yeast to human [52], which has gradually emerged as an essential regulator of gene expression homeostasis in eukaryotes at multiple levels [53], including transcription and post-transcription [50]. Likewise, recent study showed a crucial role for the chromatin remodeler SWI/SNF in regulation of splicing meiotic transcripts in *Saccharomyces cerevisiae* [54]. The other SWI/SNF complex SMARCAD1 as a transcriptional coactivator can be recruited to promoters of many genes, resulting in regulation of the target genes expression [55], whose function is involved in end resection, recombinational DNA repair and renders cells hypersensitive to DNA damage previously [56].

**Fig. 9:**
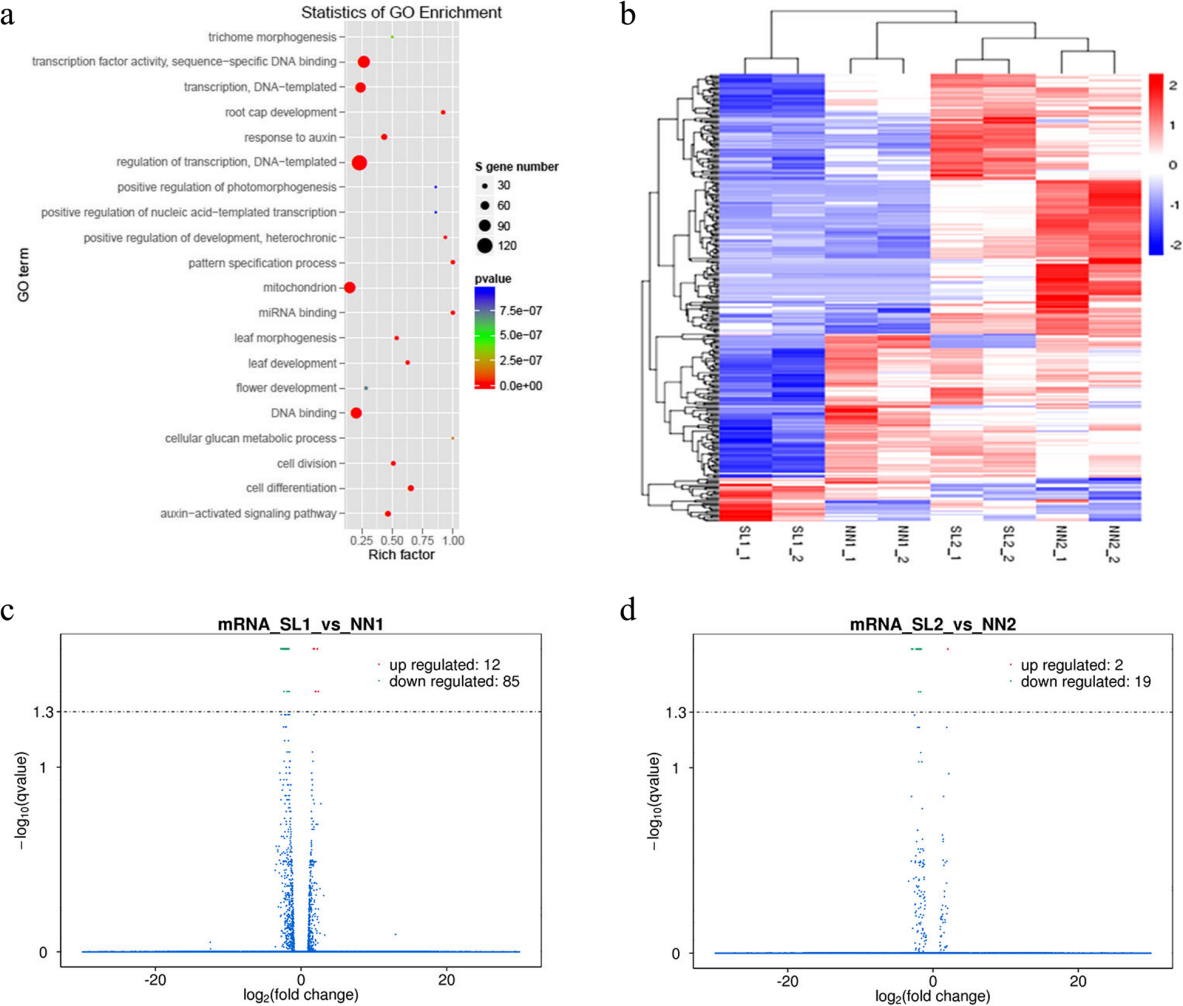
Differential expression of mRNAs between NN and SL samples. **a** The 20 most enriched pathways from GO enrichment analysis based on target genes of differentially expressed miRNAs from degradome sequencing. The *p*-values indicated the significance of the rich factor; the spot size represented the gene number. **b** Hierarchical cluster analysis of differently expressed mRNAs in the four libraries. Each sample with two biological replicates. **c** and **d** Screening of significant differentially expressed genes with Volcano chart by comparing SL1 and NN1, SL2 and NN2

The statistics of GO enrichment from degradome sequencing indicated that the GO terms of transcription and regulation of transcription showed significant difference between NN1/2 mixed library and SL1/2 mixed library (Fig. 9a). The changes that occurred in regulation of transcription pathway might be related to male sterility of 337S at SL condition. Noticeably, the expression of *SMARCA3L3* and *CAF1* was suppressed in SL plants. In order to determine whether the genes transcription in 337S were also repressed in SL plants because of *SMARCA3L3* and *CAF1* down-regulated, high-throughput mRNA sequencing were applied to detect genome-wide difference of genes expression among NN1, SL1, NN2 and SL2 samples from 337S. In general, a large number of genes were suppressed in SL samples (Fig. 9b), and many of them were significantly down-regulated, especially in SL1 compared to NN1 samples. 85 genes were significantly down-regulated but only 12 genes were up-regulated in SL1, and 19 genes were apparently down-regulated but only 2 genes were up-regulated in SL2 (Fig. 9c, d), suggesting that the regulation of transcription may be severely disrupted by the downregulation of *SMARCA3L3* and *CAF1* expression.

## Conclusion

**Fig. 10:**
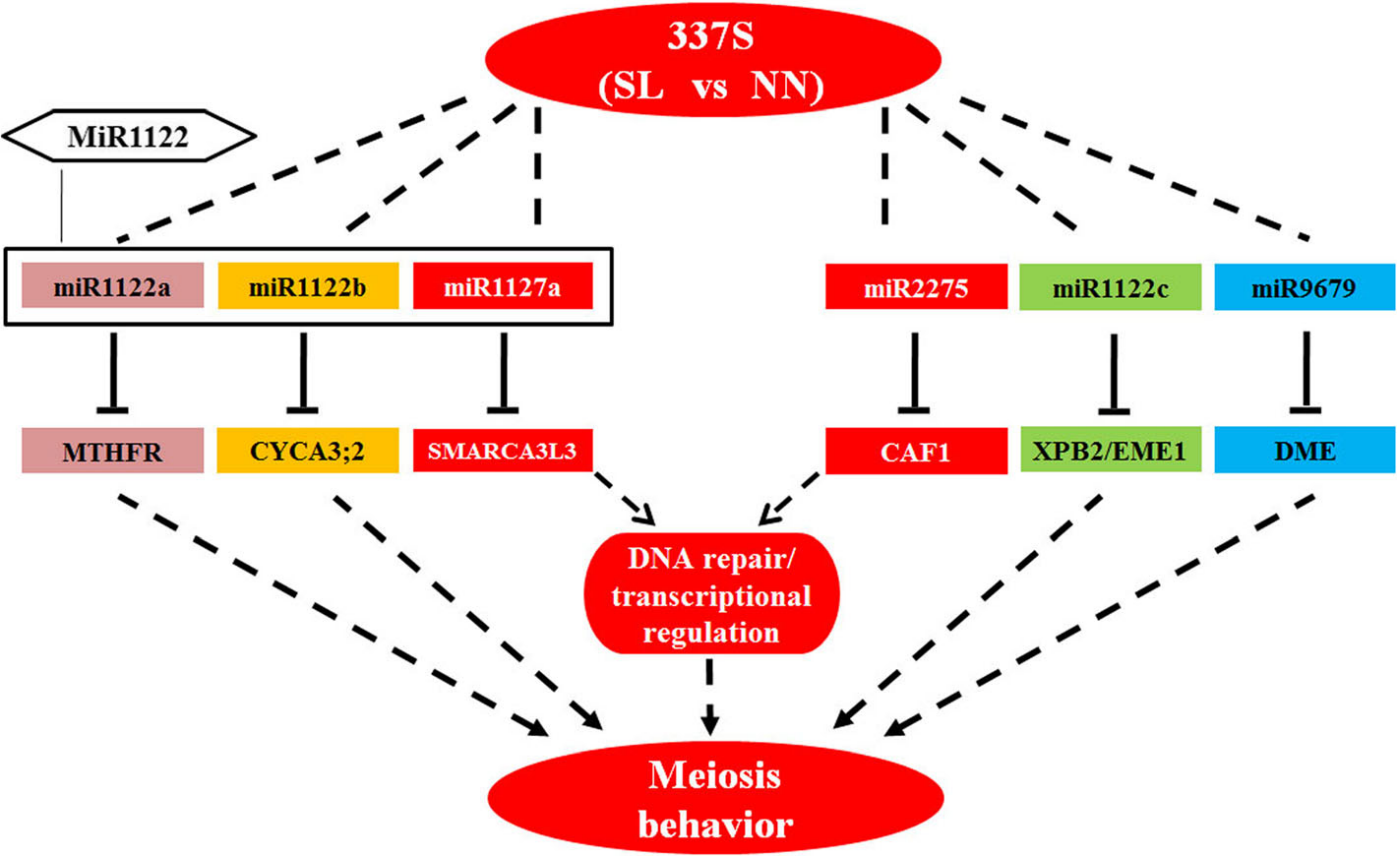
Schematic presentation of miRNA-target pairs in regulation of meiosis behavior in PTGMS line of wheat. The miRNA-target interactions of tae-miR1122a-MTHFR, tae-miR1122b-CYCA3;2, tae-miR1122c-XPB2, tae-miR1122c-EME1, tae-miR1127a-SMARCA3L3, tae-miR2275-CAF1 and tae-miR9679-DME are required for regulating male sterility in 337S at SL condition. The function of *CAF1* and *SMARCA3L3* pathway might be not only responsible for meiotic DSB repair but also for transcriptional regulation during meiosis in 337S

337S is a novel BP-PTGMS line in wheat, sensitive to both long day length/high temperature and short day length/low temperature condition. miRNAs are involved in reproductive development for many plants. tae-miR2275-3p and miRNA families of MiR1120 and MiR1122 were found to be involved in the regulation of meiosis process and early anther development in wheat. The miRNA-target interactions of tae-miR2275-CAF1 and tae-miR1127a-SMARCA3L3 might be required for regulating the progress of meiosis in male reproductive cells. The genes associated with DNA repair were altered in anthers of SL pants, implying that the failure of maintaining chromatin integrity had occurred in meiotic cells. In the meantime, the homeostasis of genome-wide genes expression might be also interfered by losing of genome stability. These results suggested that the function of *CAF1* and *SMARCA3L3* pathway might be not only responsible for transcriptional regulation but also for maintaining chromatin integrity via meiotic DSB repair in 337S. Our studies indicated that the plant regulates the faithful progression of meiosis probably via miRNA–mediated genes expression at early meiosis stages (Fig. 10). Several target genes such as *SMARCA3L3*, *CAF1*, *EME1*, *CYCA3;2*, *XPB2*, *DEMETER* and *MTHFR* whose homologues in human and yeast have been well studied in spermatogenesis and sexual reproduction, also play a critical role in anther development in wheat, indicating that the plant, mammalian and microorganism share a certain common, functionally and evolutionarily conserved regulatory mechanisms controlling meiosis behaviors.

## Additional files

Additional file 1: **Table S1.** The primers used for qRT-PCR in this study. (XLSX 12 kb)

Additional file 2: **Figure S1.** Morphological features of wheat lines HZ09, 337S and Huamai 2566 at sowing time for short day-length/low temperature condition. (JPG 216 kb)

Additional file 3: **Figure S2.** Morphological features of wheat lines HZ09, 337S and Huamai 2566 at sowing time for normal day-length/normal temperature condition. (JPG 206 kb)

Additional file 4: **Table S2.** A list of data filtering from each sample. (XLSX 11 kb)

Additional file 5: **Table S3.** Summary of known miRNAs. (XLSX 14 kb)

Additional file 6: **Figure S3.** Analysis of nucleotide bias at each position of miRNAs in NN1 (a), NN2 (b), SL1 (c) and SL2 (d) libraries. (JPG 386 kb)

Additional file 7: **Table S4.** Nucleotide sequences and read counts of identified novel miRNAs in each sample. (XLSX 13 kb)

Additional file 8: **Table S5.** The distribution of TPMs for normalized expression of miRNAs in each sample. (XLSX 11 kb)

Additional file 9: **Table S6.** The normalized expression with TPMs for all known and novel miRNAs in all samples. (XLSX 20 kb)

Additional file 10: **Figure S4.** Venn charts of differentially expressed miRNAs between SL1 vs NN1 and SL2 vs NN2. (JPG 34 kb)

Additional file 11: **Table S7.** Details of differentially expressed known and novel miRNAs in NN1 and SL1 plants. (XLSX 12 kb)

Additional file 12: **Table S8.** Details of differentially expressed known and novel miRNAs in NN2 and SL2 plants. (XLSX 12 kb)

Additional file 13: **Table S9.** The miRNAs were divided into 6 categories based on expression pattern at MMC and MP stages. (XLSX 20 kb)

Additional file 14: **Figure S5.** Fold-change of the novel miRNA in each library of 337S based on the qRT-PCR and small RNA sequencing results. (JPG 109 kb)

Additional file 15: **Table S10.** Summary data of degradome sequencing. (XLSX 11 kb)

Additional file 16: **Table S11.** List of all identified target genes for miRNAs from degradome sequencing. (XLSX 158 kb)

Additional file 17: **Table S12.** List of identified targets of differentially expressed miRNAs which were obtained from comparative analysis of SL1 and NN1, SL1 and NN1 together. (XLSX 53 kb)

Additional file 18: **Figure S6.** The expression profile of tae-miR1122c-3p targeted gene *EME1*. (JPG 35 kb)

Additional file 19: **Figure S7.** The relative expression of selected targets from degradome data for miR156 (*SPL17*), miR159 (*GAMYB*), miR160 (*ARF18*), miR164 (*CUC2*), miR167 (*ARF12*) and miR1127b (*DAD1*). (JPG 140 kb)

Additional file 20: **Table S13.** Conserved miRNAs during reproductive development in monocot and dicot plants. (XLSX 11 kb)

Additional file 21: **Table S14.** List of target genes for tae-miR9652-5p predicted by PsRobot software. (XLSX 12 kb)

Additional file 22: **Figure S8.** Expression changes of tae-miR9652-5p and its target *MEL1* at different anther development stages. (JPG 105 kb)

## Abbreviations

BP-PTGMS: Bi-pole-photo-thermo-sensitive genic male sterile
*CAF1*: *CCR4-associated factor 1*
CCR4-NOT: Carbon Catabolite Repressed 4- Negative on TATA-less
CMS: Cytoplasmic Male Sterile
*CYCA3;2*: *cyclin A3;2*
*DAD1*: *Defective in Anther Dehiscence1*
*DME*: *DEMETER*
DSBs: DNA double strand breaks
HR: Homologous recombination
*MEL1*: *Meiosis arrested at leptotene1*
miRNAs: MicroRNAs
MMC: Microspore mother cell
MP: Meiotic prophase
MTHFR: Methylenetetrahydrofolate reductase
NHEJ: Non-homologous end-joining
NN: long day length/high temperature
PTGMS: Photoperiod-Thermo-Sensitive Genic Male Sterile
*RPL23*: *60S ribosomal protein L23*
SL: Short day length/low temperature
*SMARCA3L3*: *SWI/SNF-related matrix-associated actin-dependent regulator of chromatin subfamily A, member 3-like 3*
TGMS: Thermo-sensitive genic male sterile
