# Supplementary figures and images for: Transcriptome analysis reveals new microRNAs-mediated pathway involved in anther development in male sterile wheat
Source: BMC Genomics. 2018 May 8;19:333. doi: 10.1186/s12864-018-4727-5 (PMC5941544; doi:10.1186/s12864-018-4727-5)

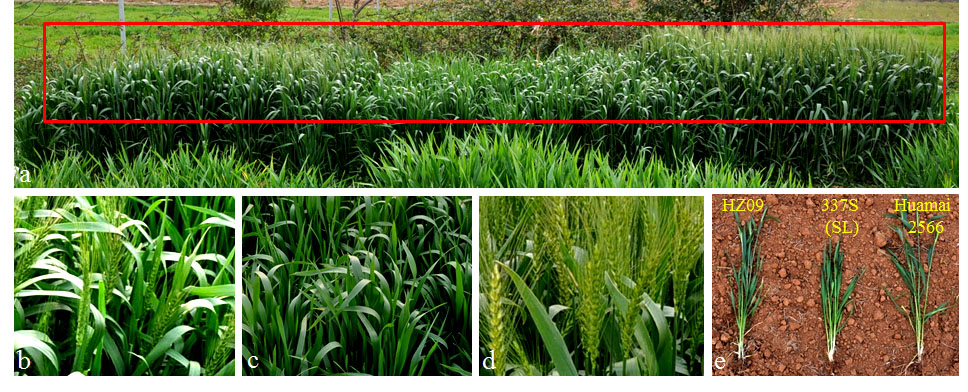

Supplement: Supplementary file 2 — Figure S1. Morphological features of wheat lines HZ09, 337S and Huamai 2566 at sowing time for short day-length/low temperature condition. (JPG 216 kb) [file 12864_2018_4727_MOESM2_ESM.jpg]

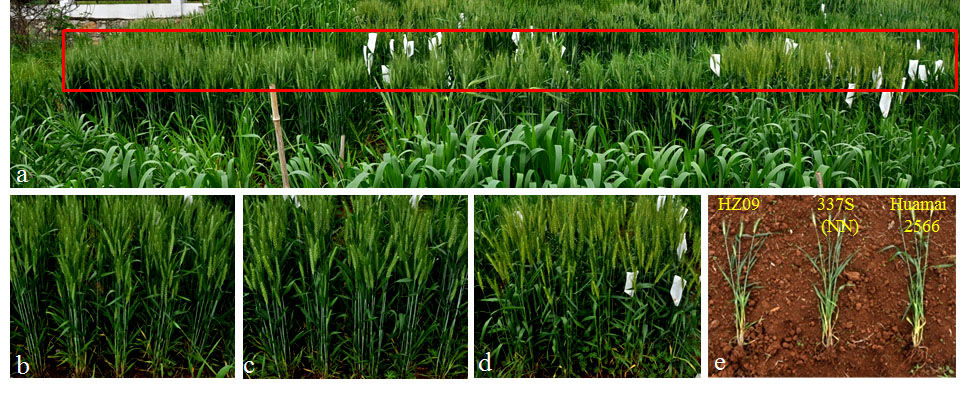

Supplement: Supplementary file 3 — Figure S2. Morphological features of wheat lines HZ09, 337S and Huamai 2566 at sowing time for normal day-length/normal temperature condition. (JPG 206 kb) [file 12864_2018_4727_MOESM3_ESM.jpg]

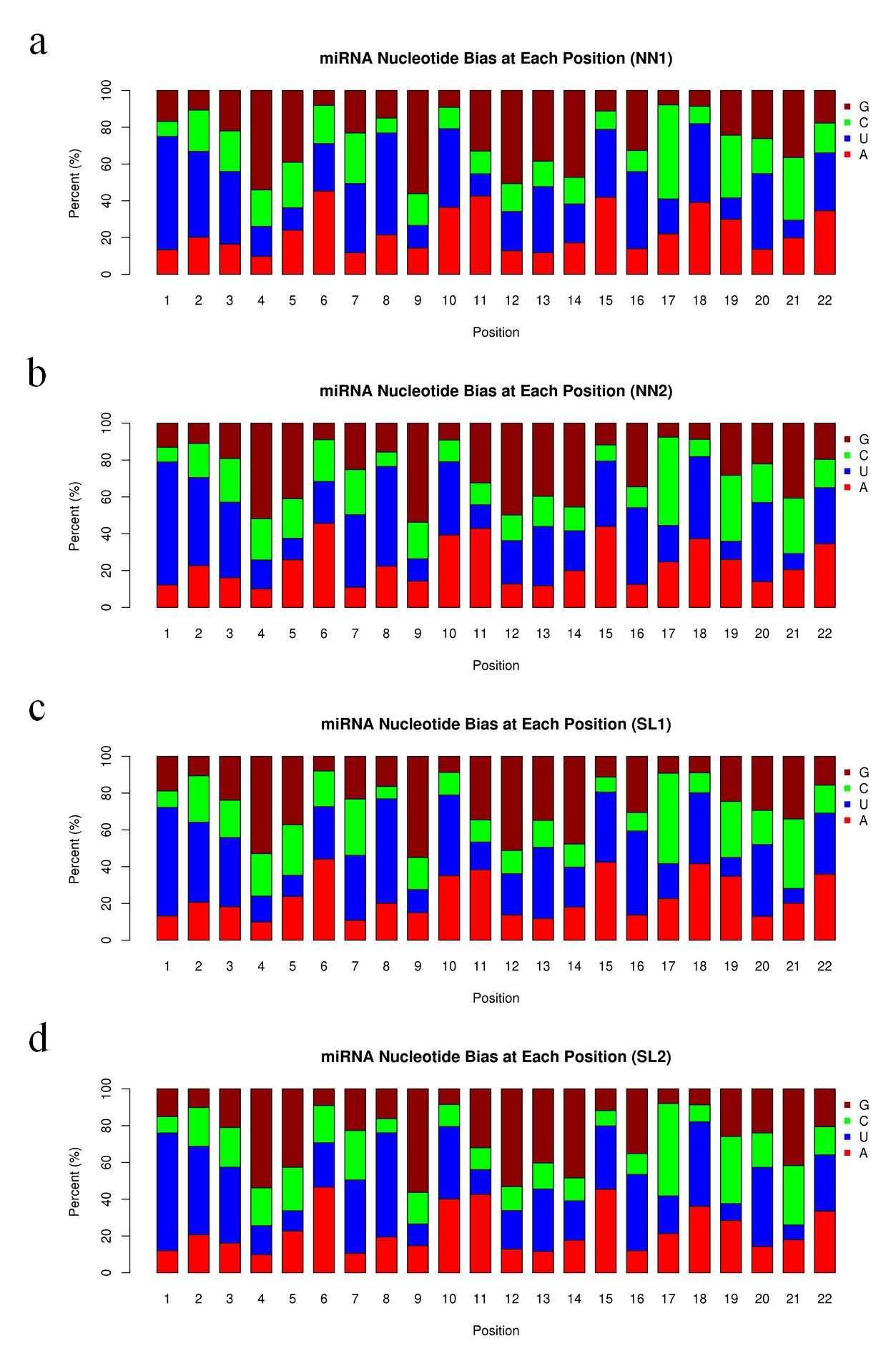

Supplement: Supplementary file 6 — Figure S3. Analysis of nucleotide bias at each position of miRNAs in NN1 (a), NN2 (b), SL1 (c) and SL2 (d) libraries. (JPG 386 kb) [file 12864_2018_4727_MOESM6_ESM.jpg]

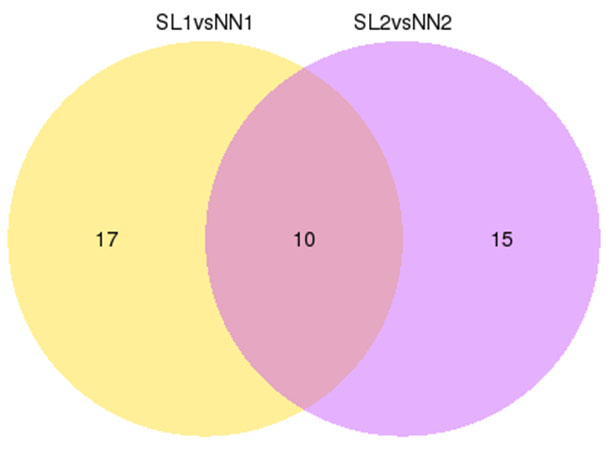

Supplement: Supplementary file 10 — Figure S4. Venn charts of differentially expressed miRNAs between SL1 vs NN1 and SL2 vs NN2. (JPG 34 kb) [file 12864_2018_4727_MOESM10_ESM.jpg]

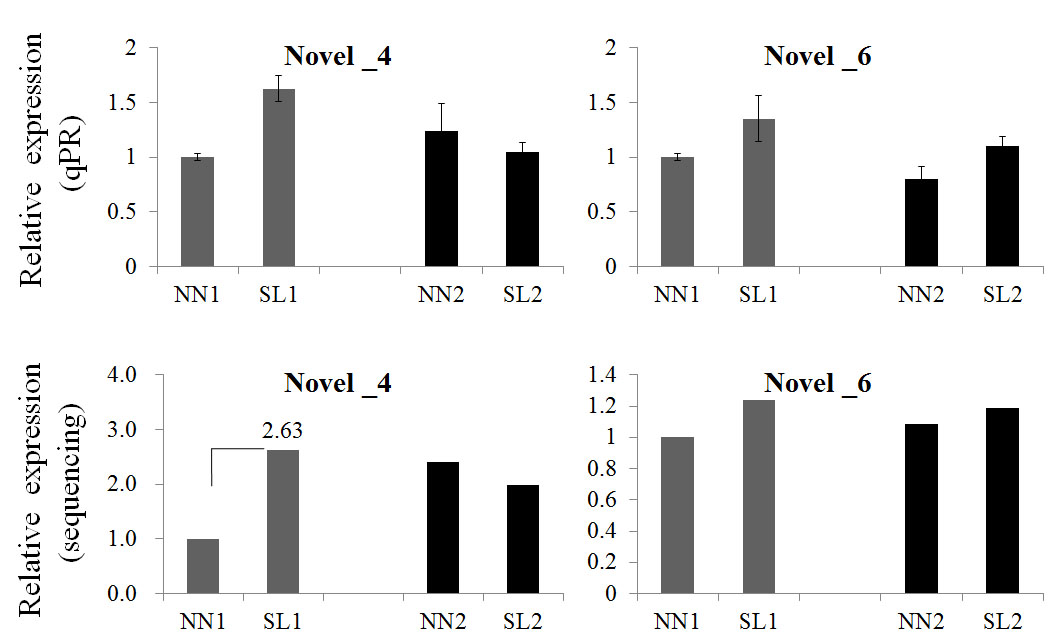

Supplement: Supplementary file 14 — Figure S5. Fold-change of the novel miRNA in each library of 337S based on the qRT-PCR and small RNA sequencing results. (JPG 109 kb) [file 12864_2018_4727_MOESM14_ESM.jpg]

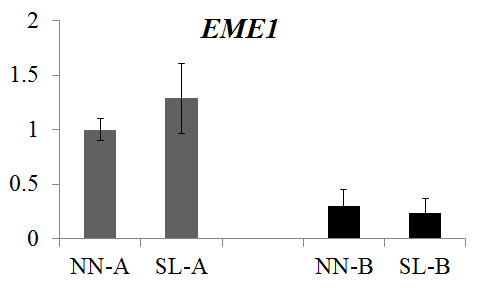

Supplement: Supplementary file 18 — Figure S6. The expression profile of tae-miR1122c-3p targeted gene EME1. (JPG 35 kb) [file 12864_2018_4727_MOESM18_ESM.jpg]

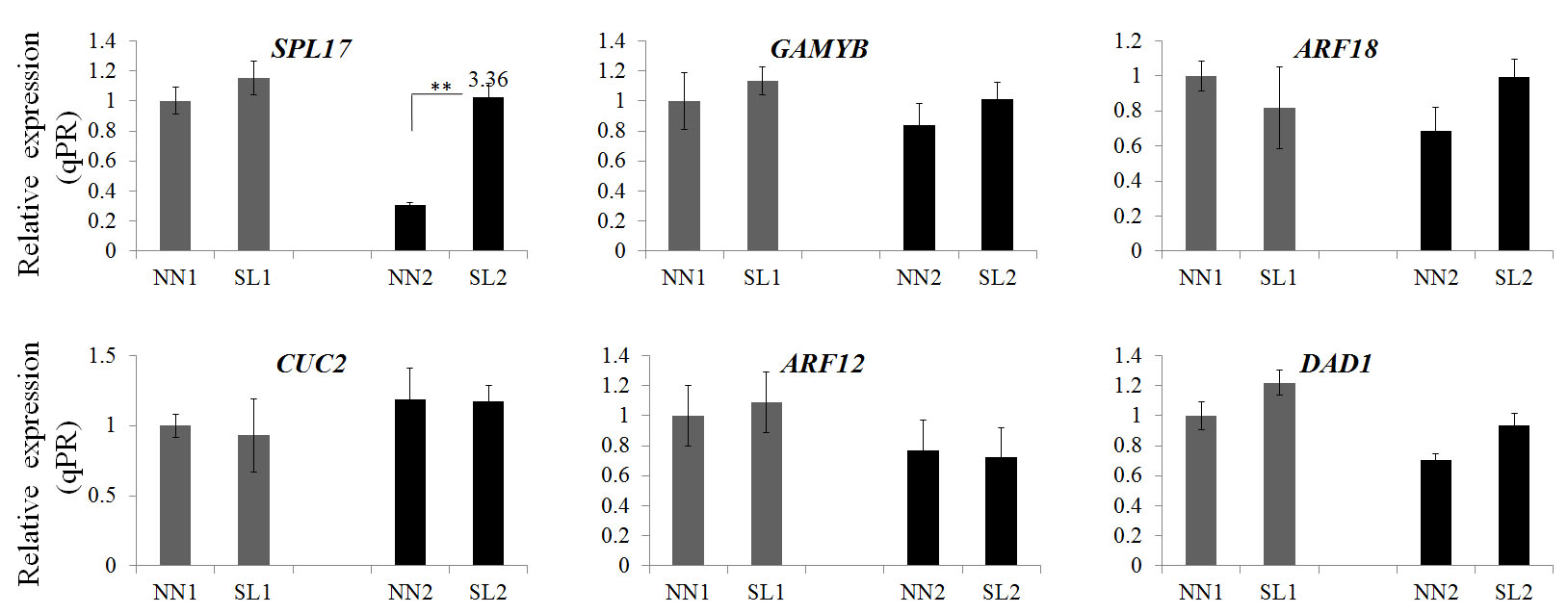

Supplement: Supplementary file 19 — Figure S7. The relative expression of selected targets from degradome data for miR156 (SPL17), miR159 (GAMYB), miR160 (ARF18), miR164 (CUC2), miR167 (ARF12) and miR1127b (DAD1). (JPG 140 kb) [file 12864_2018_4727_MOESM19_ESM.jpg]

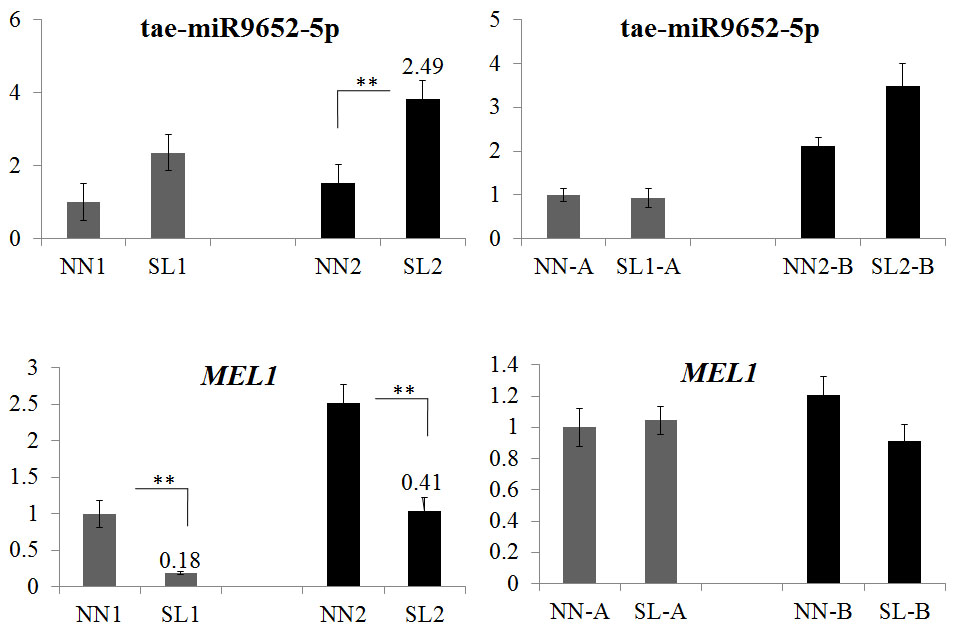

Supplement: Supplementary file 22 — Figure S8. Expression changes of tae-miR9652-5p and its target MEL1 at different anther development stages. (JPG 105 kb) [file 12864_2018_4727_MOESM22_ESM.jpg]
